# 1D and 2D annotation enrichment: a statistical method integrating quantitative proteomics with complementary high-throughput data

**DOI:** 10.1186/1471-2105-13-S16-S12

**Published:** 2012-11-05

**Authors:** Juergen Cox, Matthias Mann

**Affiliations:** 1 Department for Proteomics and Signal Transduction, Max-Planck Institute of Biochemistry, Am Klopferspitz 18, D-82152 Martinsried, Germany

**Keywords:** quantitative proteomics, high resolution mass spectrometry; genomics, proteome-transcriptome comparison, annotation analysis, non-parametric tests, false discovery rate, gene ontology, protein complexes, pathway analysis

## Abstract

Quantitative proteomics now provides abundance ratios for thousands of proteins upon perturbations. These need to be functionally interpreted and correlated to other types of quantitative genome-wide data such as the corresponding transcriptome changes. We describe a new method, 2D annotation enrichment, which compares quantitative data from any two 'omics' types in the context of categorical annotation of the proteins or genes. Suitable genome-wide categories are membership of proteins in biochemical pathways, their annotation with gene ontology terms, sub-cellular localization, the presence of protein domains or the membership in protein complexes. 2D annotation enrichment detects annotation terms whose members show consistent behavior in one or both of the data dimensions. This consistent behavior can be a correlation between the two data types, such as simultaneous up- or down-regulation in both data dimensions, or a lack thereof, such as regulation in one dimension but no change in the other. For the statistical formulation of the test we introduce a two-dimensional generalization of the nonparametric two-sample test. The false discovery rate is stringently controlled by correcting for multiple hypothesis testing. We also describe one-dimensional annotation enrichment, which can be applied to single omics data. The 1D and 2D annotation enrichment algorithms are freely available as part of the Perseus software.

## Introduction

Mass spectrometry-based proteomics can now deliver highly accurate data on hundreds of thousands of peptide features in a single biological project[1]. Accurate comparative protein quantification is feasible for thousands of proteins with methods based on stable isotopic labeling[2] but also in label free approaches[3]. Coverage of these methods has already reached complete proteome scale for the yeast model organism[4] and other proteomes of similar or lower complexity. With further improvements in the underlying technologies comprehensive quantification in human cells seems also likely to be achieved soon. Therefore one can now carry out quantitative expression proteomics on a similar scale as for nucleic acids with microarrays or more recently with deep sequencing approaches[5].

The ability to perform side-by-side large-scale quantitative profiling of the proteome ,transcriptome or genome raises the question which classes of gene products show concordant and which show discordant behavior between the different levels of gene expression. For instance, one general question in proteomics is how far absolute levels of expression changes correlate between the transcriptome and the proteome. In the hypothetical case of pure transcriptional regulation the correlation between these two levels would be near one, and would only be limited by the technical limitations and imperfections of the respective quantitative profiling technologies. Indeed, while early investigations found low or no correlation between proteome and transcriptome[6,7], recently much higher levels of global correlation have been reported[8,9].

While transcriptional regulation is generally a dominant aspect of the entire expression cascade, there are many known examples of posttranscriptional regulation like micro-RNA controlled inhibition of transcripts[10] and directed protein degradation[11]. Therefore it would be desirable to have a method that highlights those proteins or protein classes for which a differential behavior is observed at different levels of the gene expression program. A straightforward manual approach is to make a scatter plot of protein changes versus transcript changes and look for single 'off-diagonal data points' or whole 'off-diagonal data point clouds'. This can be very instructive and is indeed recommended as a first step of data analysis. However, this quickly becomes very complex and in any case it is desirable to have formal statistical criteria for assigning 'interestingness' to protein classes in this scatter plot. As an example one may want to have a p-value based approach for the question whether the points in the scatter plot that correspond to proteins in the proteasome core complex show a significantly different distribution from all the other proteins. Furthermore, if that is the case we would like to have some sort of score for the proteasome that describes the direction in the scatter plot in which the proteasome components tend to deviate from the overall distribution of all proteins. This directional score will then indicate whether the statistically significant behavior of the peoteasomal proteins is the same between transcriptome and proteome or if it is 'off-diagonal' i.e. differing between the transcriptome and the proteome. Finally, in case we repeat the p-value calculation over many complexes or other annotation terms we need to take precautions for multiple hypothesis testing. All the issues described above are addressed by the 2D annotation enrichment method introduced in this manuscript. In the following sections we describe all the details of the method, illustrate its principles with relevant examples and give details on how readers can apply it to their own data.

## Materials and methods

The protein intensity data used in the explanation of the 1D annotation distribution in the Results section is taken from a label-free proteome study of mouse dendritic cells to a depth of 5,780 proteins[3]. In that study, cell sub-populations were obtained by Fluorescent Activated Cell sorting (FACS), proteins were separated by 1D SDS PAGE and digested with trypsin.

The yeast data used in the sub-section on 2D annotation enrichment is obtained from de Godoy et al.[4] where haploid and diploid cells were differentially labeled with Lys8/Lys0 SILAC[2]. The microarray data for the comparison is from Galitski et al[12]. The data was filtered as described in[4]. For the proteome versus DNA copy number example, the proteomic as well as the comparative genomic hybridization (array CGH) data is from Geiger et al.[13]

In all cases peptides were analyzed on a nanoflow HPLC system connected to a hybrid LTQ-Orbitrap or Orbitrap-Velos mass spectrometer (Thermo Fisher Scientific). Human and mouse data were searched against International Protein Index[14] (IPI) protein sequences while the yeast data was searched against the protein translations in the Saccaromyces Genome Database[15] (SGD).

## Results

We start the description of the data analysis workflow at the point where protein abundances or protein expression ratios have already been calculated. While all examples show proteomics data obtained with the MaxQuant software[16,17] in combination with the Andromeda search engine[18], this is not a prerequisite for the application of the 2D annotation enrichment method. In fact, the quantitative proteomics data could have been produced by any other software or search engine. Similarly, data from other levels of expression are not limited to any particular technology or software platform. Ideally the measurements from the different technologies are done on the same samples, but this is not strictly necessary.

When reporting quantitative data for proteins from shotgun proteomics data, care has to be taken in the counting of independent protein identifications. The measured peptides may in some cases not be unique and map to several proteins, which is called the protein interference problem[19]. Only proteins that are distinguishable based on the measured set of quantified peptides should be counted as individual protein identifications. All redundancy on the protein level should be removed, for example by collapsing them to protein groups. This is a particularly important point when performing annotation enrichment analysis. For instance, assume that there are ten isoforms of a particular protein reported as independent protein identifications even though the quantified peptides for all of them are the same. If one would keep all ten isoforms as separate identified proteins their quantitative profiles would perfectly correlate and their annotation terms would be highly similar. This would obviously lead to irresolvable difficulties in statistical tests for contingency between the quantitative expression data and the annotation terms and would likely produce false positives. Hereafter we always refer to suitable groups of proteins in the above sense when we use the word protein. MaxQuant automatically performs this grouping and is furthermore integrated with subsequent bioinformatic analysis in the freely available Perseus software[20] which implements all algorithms described below.

### Matching proteins to other high-throughput data

When using MaxQuant, if several quantitative experiments are combined or replicates were made these will all be projected onto the same protein grouping over all 'quantitative columns'. Therefore, the proteome data will be in a convenient matrix form already, even for very complex experimental designs. This will not be the case when one wants to compare the proteome data with transcriptome data, for instance. Several probe sets of an Affymetrix chip measure the same gene and there may be several genes belonging to the same protein group. For the matching we take a protein centric view. For each protein in the protein group we determine all probe sets that are annotated in the chip annotation file with a UniProt identifier. It is not trivial to decide which UniProt identifiers to use for a group of proteins that are indistinguishable by the measured peptides. A protein group consists of proteins from the list of protein sequences submitted to the search engine that cannot be quantified independently based on the set of identified peptides. In particular, if two proteins have identical sets of identified peptides they will be grouped together. Also if the set of identified peptides of one protein is completely contained in the set of identified peptides of another protein, these two proteins will be combined in a protein group as well. Proteins within a protein group are sorted by the number of identified peptides in descending order. For the remaining ambiguities we use the razor peptide or parsimony concept, which means that the peptide is assigned according to Occam's razor principle to the protein group that most plausibly explains its existence, which is the one which already has the most peptide identifications assigned to it.

The number of probe sets matched in this way to every protein group can vary now from zero to several. If none is matching then no comparison can be made for this protein. If one is matching, then the quantitative information for this probe set will be used. If several probe sets match the point-wise median of their quantitative profiles is taken. Expression data from other microarray types can be matched in a similar way as long as the vendor provides UniProt or other protein identifiers for the hybridization probes. Deep RNA sequencing data is also easily matched, for instance in the form of RPKM values[21] produced by a suitable software. DNA copy numbers from comparative genomic hybridization[22] are associated with the closest protein coding gene for each hybridization probe. All copy numbers that have the same nearest protein coding gene are combined by taking their median value.

. Note that for the quantitative analysis of expression data (irrespective of which kind) it is usually advisable to take the logarithm before proceeding with further steps. This is true for ratios as well as for abundance data. Also here before averaging expression profiles this is advisable, even if the median is taken. This is because also for the median an averaging can take place between the two central numbers in case there is an even number of values. The need for taking logarithms becomes immediately apparent in the case of ratios. One would expect that the average of a two-fold up-regulation and a two-fold down-regulation should be no regulation. This is however not the case if the ratios are averaged (2 + 1/2)/2 = 1.25 ≠ 1. If logarithms (e.g. to the base two) are averaged the desired result is obtained: (log(2) + log(1/2))/2 = 0. The base of the logarithm does not matter in principle since it can be absorbed in an overall factor multiplied to all the data. However it is customary to use base two for ratio data and base two or ten for abundance data.

### Protein annotations

We base the annotation of proteins on UniProt identifiers[23]. Also here, as was the case for the matching of proteins to other 'omics' data, one has to take care of the selection of UniProt identifiers for a particular protein group. Since each of the proteins in a protein group potentially can have different annotation terms it is a non-trivial question which terms should be reported for the protein group as a whole and then be used in the enrichment analysis. Selecting only the annotation of the protein with the most peptides (which is anyway not necessarily a unique choice) might not always be the best decision since only a shorter or 'activated' version of the protein might carry the complete annotation. On the other hand, including the annotation of all protein group members may pick up false positives because there may be proteins in the group only due to a single peptide and not because of a genuine relatedness of the protein. We found that a good compromise is to include the annotation of all proteins in the protein group that have at least half the number of peptides of the leading protein which has the maximal number of peptides in the group. This choice usually avoids the two pitfalls mentioned above.

One major source of annotation is the gene ontology[24] (GO) which carries information on biological processes, molecular functions and sub-cellular localization. We use the Kyoto Encyclopedia of Genes and Genomes (KEGG) database[25] as a source for pathway membership information and we infer the protein domain content from the Pfam database[26]. For human data the Corum database[27] is a well curated repository of protein complexes. Expression in tissues (as a yes/no information) can also be included as annotation. Annotation relating to the transcripts or genes can also be included. For instance miRNA binding sites in the three-prime region of the transcript can be treated as categorical annotation. Even knockout phenotypes, if they exist for the particular organism, can be treated as an annotation. Indeed any kind of information that can be summarized in terms that can be ascribed to proteins can serve as a source of annotation, whose enrichment in the process under study can be tested.

### 1D annotation enrichment

The 2D annotation enrichment algorithm works equally well for 1D data, such as any quantitative proteomics experiment. We first describe the principle of the 1D distribution analysis, which also serves as a preparation for the 2D algorithm. The input is a single column of numerical values assigning one numerical value to every protein. These values are typically protein ratios or absolute protein abundances. They could also be derived quantities, like average fold-changes between replicate groups or p-values or test statistic resulting from a test for significant changes in protein expression. If the column has missing values then the respective proteins will be ignored in further analysis.

**Figure 1:**
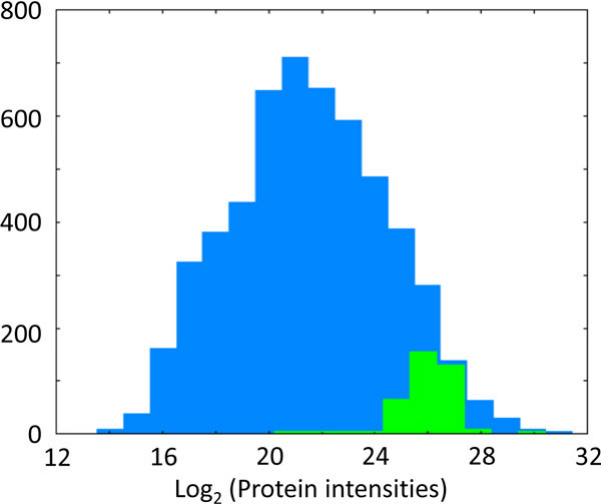
**Histogram of log protein intensities for all mouse proteins quantified in dendritic cells in Luber et al**[3]**(blue)**. The green histogram indicates the ribosomal proteins within this distribution. They are significantly enriched at large values. Heights of the green bars were multiplied by five for better visibility.

We wish to test for every annotation term (such as every protein complex or pathway) whether the corresponding numerical values have a preference to be systematically larger or smaller than the global distribution of the values for all proteins. In the schematic example displayed in Figure 1 the total distribution of all log protein abundances in the mouse dendritic proteome is shown in blue whereas proteins belonging to the Gene Ontology Cellular Component (GOCC) categorization 'ribosome' are displayed as a green histogram. To test if the ribosomal proteins are statistically significantly enriched at high protein abundance values, we perform a two-sample test for difference of means, where one group consists of all proteins that are categorized as ribosomal and the other group contains all remaining proteins. In our example of the ribosomal proteins only testing for enrichment at large values makes sense, but in general enrichment of annotation terms at small values will also be of interest. For this purpose we perform a two-sided test which results in significance if the protein category is significantly enriched at large or at small values. (If desired, the respective one-sided tests can also be performed in the software implementation.)

To be independent of the shape of the distribution we apply a non-parametric test which in particular does not assume a normal distribution of the numerical values. These properties single out the two-sided (two-sample) Wilcoxon-Mann-Whitney test as the method of choice, which we apply to all protein categorizations in a given set of terms.

The Wilcoxon-Mann-Whitney test assesses whether one of two observation groups tends to have larger values than the other. The method works entirely with the ranking of the values and checks in our case if the proteins of interest tend to be ranked higher (or lower) as a group relative to the ranking of all proteins. The test statistic is

$$R_{1}-\frac{n_{1}\left(n_{1}+1\right)}{2}$$

Where *n*_1 _is the size of group 1 and *R*_1 _is the sum of ranks in group 1.

Technically, the Mann-Whitney test assumes independence of the values, which is a good approximation in our case, in particular since every peptide is used in only one protein group for quantification. If non-unique peptides were used in several protein groups, the independence assumption would not hold.

The number of terms and therefore also the number of hypotheses tested simultaneously can be quite large. For instance, there are 9,732 different terms among the GO molecular functions. This makes it important to adjust for multiple hypothesis testing. We apply the Benjamini-Hochberg method[28] and based on experience with the results we employ by default a false discovery rate of 2%, however this is an adjustable parameter that can be set by the user. For those categories that are significant we calculate a position score indicating where the center of the distribution of values for the protein category is located relative to the overall distribution of values. This score is defined as

$$\text{s = 2(}{\text{R}}_{\text{1}}-{\text{R}}_{\text{2}})/\text{n}$$

where R_1 _and R_2 _are the average ranks within the group under consideration and its complement (all remaining proteins in the experiment), respectively and n is the total number of data points. It is a number between -1 and 1. A value near 1 indicates that the protein category is strongly concentrated at the high end of the numerical distribution while a value near -1 means that the values are all at the low end of the distribution. For significant terms it is not possible that s reaches zero exactly, but especially for larger categories that show a slight but consistent trend it is possible to have small absolute values of s. A moderately positive value of s for a category with many members, for instance, indicates that there is a significant collective shift towards larger values for this category which however is small in absolute terms and possibly not noticeable when looking at individual proteins. Note that the method's calculations are entirely based on information within the measured proteome. Often, enrichment calculations in proteomics against the whole genome are problematic. By construction these problems are completely circumvented here.

In the ribosome example of Figure 1, the p-value is 2 × 10^-37^ and the s-value is 0.85, indicating that ribosomal proteins are strongly enriched among the most abundant proteins.

When applied to ratios of protein abundances the method described here is similar to the quantile-based enrichment calculations introduced by Pan et al.[29] There the distribution of protein ratios was subdivided into bins and then all categories were tested for being enriched in these bins. In contrast, the 1D annotation enrichment developed here has the advantage that it is not necessary to define a somewhat arbitrary positioning of bin boundaries beforehand. Instead the distribution of values is scanned for interesting sub-categories in an unbiased way without using thresholds.

### 2D annotation enrichment

**Figure 2:**
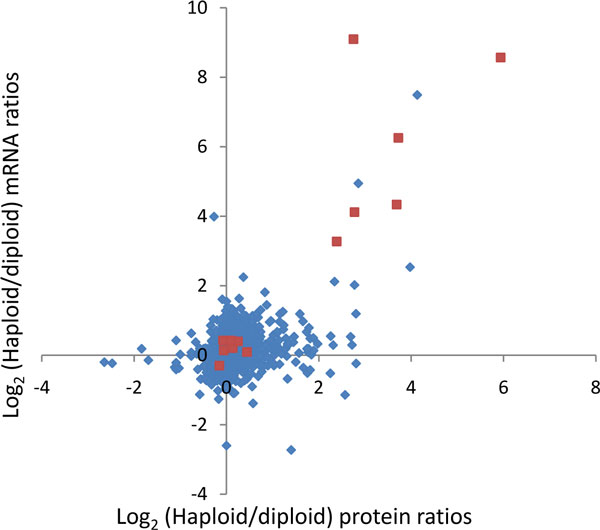
**Yeast protein ratios vs. mRNA ratios between the haploid and diploid populations from de Godoy et al**[4]. The data points in red belong to the Gene Ontology (GO) biological process 'pheromone-dependent signal transduction during conjugation with cellular fusion'.

For the analysis of quantitative protein expression values together with other high throughput data we would like to generalize the method described above to the joint distribution of two numerical quantities. To be specific in the further discussion we will assume that the other high throughput data to be analyzed together with proteomic data is constituted by mRNA expression levels. One may for instance be interested in the enrichments of annotations in the plane spanned by protein abundances and mRNA abundances. Similarly one may wish to plot protein abundance ratios (e.g. from isotopic labeling experiments) against mRNA abundance ratios between the same samples. Figure 2 displays an example where protein abundance ratios between haploid and diploid yeast cells are plotted against the corresponding mRNA ratios. It may also be of interest to compare p-values for significant changes from a proteomics experiment with p-values from the corresponding mRNA based measurement series on the same samples. Any quantitative values that are comparable at the proteomics and mRNA (or other -omics) level and that are derived from the same or similar samples can be used.

Also for the two-dimensional case, we want to avoid the normality assumption and therefore wish to use a non-parametric testing strategy. What is needed for the generalization to two numerical dimensions is a replacement of the Wilcoxon-Mann-Whitney test that works with two-dimensional input data. All the remaining strategy can then be taken over from the one-dimensional case. The concept of rank sums that is used in the definition of the test statistic for the Wilcoxon-Mann-Whitney test at first appears to be tied to the one-dimensional case since only in the one-dimensional case is it possible to define an order of the data points in a meaningful way. For points in a two-dimensional plane, in contrast, a natural order relationship does not exist. The situation is different for parametric tests, like Student t-test or analysis of variance (ANOVA) where the generalization to the multivariate case is straightforward and known as multivariate analysis of variance (MANOVA) (see e.g. reference[30]), which is is a statistical test procedure for comparing multivariate population means of several groups. To solve our problem of non-parametric tests in higher dimensions, we make use of the circumstance that the Wilcoxon-Mann-Whitney test is equivalent to the simple Student t-test performed on the ranks[31]. This is the case because the t-test statistics calculated from the ranked data is a monotone function of the rank sum expression that is used for the Wilcoxon-Mann-Whitney test statistic. Combining the fact that the non-parametric test is equivalent to the parametric test on ranks and that ANOVA has a straightforward generalization to higher dimensions, we propose to use the MANOVA test on the ranked multivariate data, where the data is replaced by ranks in each dimension separately.

The test statistic for the MANOVA test for two groups in two dimensions are given here for reference. It is proportional to

$$\frac{s_{xx}d_{y}^{2}+s_{yy}d_{x}^{2}-2s_{xy}d_{x}d_{y}}{s_{xx}s_{yy}-s_{xy}^{2}}$$

where

$$d_{x}={\bar{x}}_{1}-{\bar{x}}_{2}\;\text{and}\;d_{y}={ȳ}_{1}-{ȳ}_{2}$$

are the differences of the group means between group 1 and 2 in the x and y coordinartes, respectively,

$$\begin{array}{c} s_{xx}=\overset{n_{1}}{\underset{j=1}{\sum }}{\left(x_{1,j}-{\bar{x}}_{1}\right)}^{2}+\overset{n_{\text{2}}}{\underset{j=1}{\sum }}{\left(x_{2,j}-{\bar{x}}_{2}\right)}^{2}\\ s_{yy}=\overset{n_{1}}{\underset{j=1}{\sum }}{\left(y_{1,j}-{ȳ}_{1}\right)}^{2}+\overset{n_{\text{2}}}{\underset{j=1}{\sum }}{\left(y_{2,j}-{ȳ}_{2}\right)}^{2}\\ s_{xy}=\overset{n_{1}}{\underset{j=1}{\sum }}\left(x_{1,j}-{\bar{x}}_{1}\right)\left(y_{1,j}-{ȳ}_{1}\right)+\overset{n_{2}}{\underset{j=1}{\sum }}\left(x_{2,j}-{\bar{x}}_{2}\right)\left(y_{2,j}-{ȳ}_{2}\right) \end{array}$$

are the summed squares of the deviations from the group means for x, y and mixed coordinates,

$${\bar{x}}_{1},{\bar{x}}_{2},{ȳ}_{1},\;\text{and}\;{ȳ}_{2}$$

are the means of groups 1 and 2 in x and y coordinates,

$$n_{1}\;\text{and}\;n_{\text{2}}$$

are the sizes of groups 1 and 2 and

$${\bar{x}}_{1,j},{\bar{x}}_{2,j},{ȳ}_{1,j},\;\text{and}\;{ȳ}_{2,j}$$

are the ranked values for x and y dimensions, separated into group 1 and 2.

We define the resulting MANOVA test result as the 2D annotation enrichment p-value. The FDR of this approach can be controlled with the Benjamini-Hochberg method in the same way as was done for the one-dimensional case.

**Figure 3:**
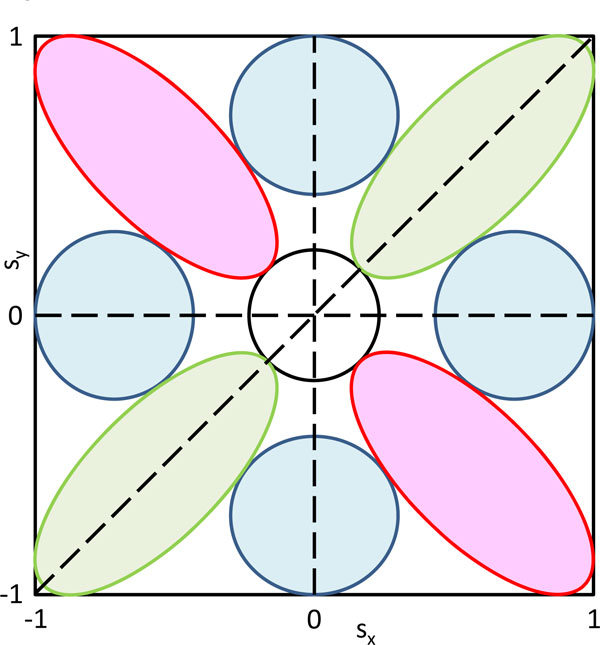
**Schematic representation of the 2D annotation enrichment score**. The score is a number pair inside the displayed rectangle. Significant terms will avoid a circular region around the origin. The green regions correspond to concordant up or down regulation. The blue regions correspond to terms that are up or down in one direction, but not in the other, while the terms in the red regions show anti-correlating behavior.

After determining which annotation terms show a significantly deviating protein/mRNA level distribution, we calculate an s-score in analogy to the one-dimensional case. Now the score is a number pair (s_x_, s_y_), the coordinate-wise difference of average ranks used in the one-dimensional case. It is confined to the square -1 ≤ s_x _≤ 1 and -1 ≤ s_y _≤ 1. The point (s_x_, s_y_) = (0,0) corresponds to annotation terms that are not distributed differently from the overall distribution of value pairs. The significance cutoff creates an empty region around the origin. The remaining parts of the rectangle can be subdivided into eight regions corresponding to correlating, non-correlating and anti-correlating regions (see Figure 3). For instance, the green oval in the upper right corner contains annotation terms whose members tend to be up-regulated on protein as well as mRNA levels. Similarly, the other green oval contains terms that show correlating down-regulation on both levels. The blue regions correspond to terms that are only up or down in either protein or mRNA level, while the terms in the red regions show anti-correlating behavior between proteins and transcripts. The exact limits of the regions should not be taken literally in Figure 3 which only displays the general possible behaviors of annotation terms. The exact subdivision usually becomes clear in real examples by visual inspection of the scatter plot of the score for all significant terms. A generalization to multi-dimensions of 'omics' data is possible and will be included in later releases of the software.

**Figure 4:**
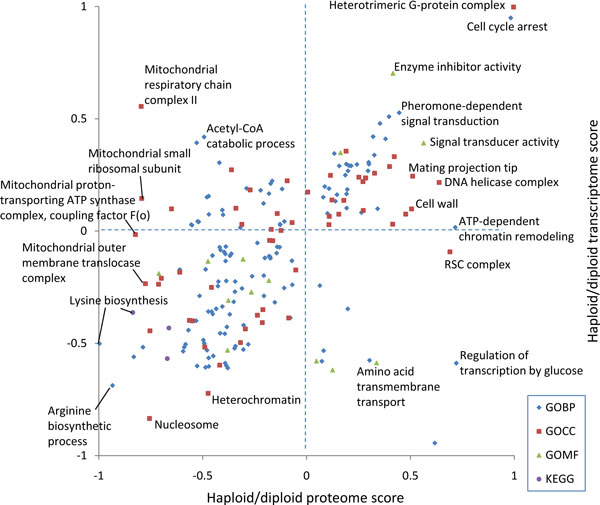
**2D annotation enrichment based on the yeast protein and mRNA ratios displayed in Figure 2**. 'Pheromone-dependent signal transduction' is located near the diagonal with positive values for both scores. 'Cell wall' has only a small mRNA score but a large protein score.

Figure 4 shows the result obtained for the haploid-to-diploid protein and mRNA ratios in yeast displayed in Figure 2. All terms which are significant with a false discovery rate of 0.15 are shown. Pheromone-dependent signal transduction is located in the quadrant where both scores are positive and can be found close to the diagonal. This indicates that components of the pheromone pathway are up-regulated in haploid cells as messages as well as proteins, as is expected from the biology of these cell types (diploid yeast cannot mate). As can be seen in Figure 2, not all members of the pheromone Gene Ontology Biological Process (GOBP) terms are up-regulated. Nevertheless, this annotation term is picked up by the 2D annotation enrichment at the chosen FDR. Another interesting example is the GOCC term 'cell wall' which is located near the line of zero mRNA score but is high-scoring in the protein direction. The abundance increase of cell wall proteins can be ascribed to the different surface-to-volume ratios of haploid and diploid cells and their ratios were even found to be consistent with geometrical considerations[4]. Here we see that this is predominantly an effect of protein abundances and that there is only a small effect on the mRNA amounts. Another interesting case are the mitochondrial protein complexes around a low proteome score of -0.8 that all have very different transcriptome scores. This indicates that the copy numbers of the proteins involved in these complexes are tightly controlled to have suitable amounts for their collaborative roles in mitochondria. Apparently, the corresponding amounts of messages are not adjusted to each other indicating that the regulation does not happen at the transcriptional stage.

**Figure 5:**
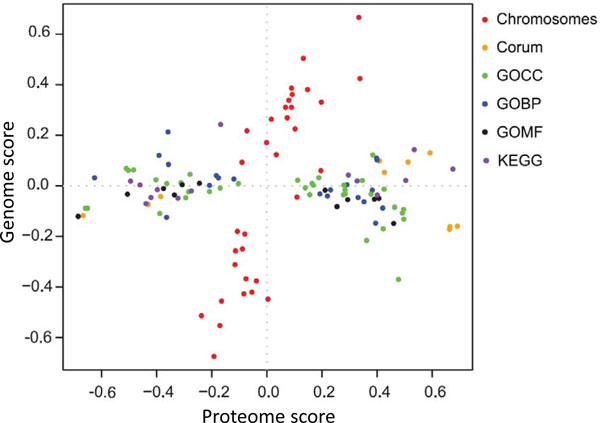
**2D annotation enrichment for Comparative Genomic Hybridization (CGH) ratios (vertical) vs. protein ratios (horizontal) from Geiger et al**[13]. Significant complexes, pathways and gene ontology terms are all distributed along the proteome change direction. Only the chromosome annotations have a major contribution in the vertical direction.

Another example is shown in Figure 5 where protein levels are compared to gene copy numbers in cancer cell lines which show a tendency towards regional copy number variations along the chromosomes. At the protein level, ratios were measured between the breast cancer cell line of interest and a similar 'normal' cell line without regional copy number variations. The corresponding copy number ratios between the same cell types were measured using comparative genomic hybridization (array CGH). In Figure 5 the significant annotation terms of the 2D annotation enrichment of the matched data is shown. In addition to the usual annotation terms the chromosome of each gene was also included (red dots). As can be seen all functional annotation terms such as biochemical pathways, protein complexes and sub-cellular localizations are extended along the proteome direction. The only terms with a major contribution in the genome direction are the chromosomes themselves. If the proteome changes had been one-to-one translations of the DNA dosage changes all terms should have been arranged along the diagonal line x = y. Obviously this is not the case, showing that substantial secondary regulation is involved in setting the final protein levels, presumably adjusting concentrations to the amounts suitable for proper functioning of pathways and complexes.

### Software implementation

**Figure 6:**
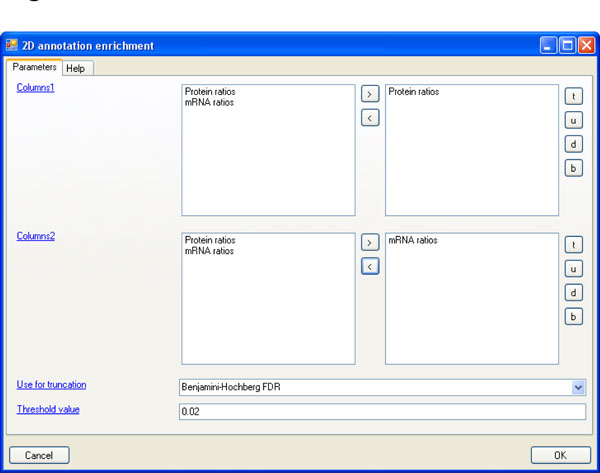
**Parameter window of the 2D annotation enrichment in the Perseus software**.

The 2D enrichment analysis is integrated into the Perseus software package which will be described elsewhere. Perseus is freely available and can be downloaded from www.biochem.mpg.de/mann/tools/. All necessary preprocessing and normalization steps can be found in the 'Processing' menu in Perseus. The 2D enrichment analysis is located in the main menu under 'Processing → Annotation → 2D annotation enrichment'. Figure 6 shows the parameter panel where values for the input parameters can be specified. The analysis can be performed on multiple pairs of 'x-axes' and 'y-axes'. The respective quantitative columns can be specified in the fields named 'Columns1' and 'Columns2'. The number of columns in these two fields must be equal and corresponding pairs of columns will be analyzed together, i.e. the first column in the first field together with the first column in the second field, the second column in the first field together with the second column in the second field, etc. If only one 2D annotation enrichment should be performed there will be only one entry in each of the two fields. The parameter 'Use for truncation' specifies that the list of significant hits should be terminated with a p-value threshold or that a Benjamini-Hochberg FDR should be applied. Under 'Threshold value' the actual value for this truncation is supplied, either the p-value or the FDR. In case of FDR it is specified as a value between 0 and 1, not as a percentage.

## Discussion

While replicates within one technology are usually best done as 'biological' as possible to ensure that the findings are robust and reproducible, for cross technology comparisons it is more desirable to have the equivalent of a 'technical' replicate. For instance, the cell populations from which the transcriptome and the proteome are measured should be as similar as possible, ideally aliquots from the same sample so that one is sure that one samples the same cellular state on different levels of expression. If desired, the whole measurement including the proteome and the transcriptome can be repeated as 'biological' replicates. In the majority of cases, however, the available data has not been recorded in this optimal way. Of course the data analysis described here can still be applied to this situation as well.

Often enrichment analysis in proteomics is performed by calculating a p-value corresponding to a test if a certain annotation term is enriched in a certain set of proteins relative to all genes in the genome. The results of this kind of calculations have to be taken with caution, especially in cases where the proteome coverage is far away from saturation or completeness since apart from the effect under investigation they are biased by which proteins are measurable at all by the employed mass spectrometric technology[32]. This may lead to seemingly significant p-values for large protein categories only because the measurable and abundant proteins tend to have more annotation. Another bias can come from which proteins are expressed at all in the proteome under study. We completely avoid this problem by basing the enrichment calculations always on the protein population that has been observed in the measurement.

Another issue of interest is the potential application of corrections when multiple related terms are used for statistical comparisons. For example, terms in GO are mutually dependent. In principle correction methods like this can be applied to 1D and 2D annotation enrichment as well and we might do so in the future. Note, however that by not taking the hierarchy and relatedness of terms into account the significant findings reported after multiple hypothesis correction are on the conservative side, since the number of effectively independent tests is lower than the total number of terms which is used in the multiple testing correction. Therefore there is no danger of over-reporting. On the contrary, at fixed FDR one might miss a few significant terms which one would have obtained with a method taking the relatedness into account.

Many other tools for enrichment analysis already exist. In Hauang et al.[33] the authors categorize existing tools into three classes: singular enrichment analysis (SEA), gene set enrichment analysis (GSEA) and modular enrichment analysis (MEA). In this kind of classification our 1D annotation enrichment belongs to the GSEA class, because it is a 'no-cutoff' method. This means that it is not necessary to define a set of regulated proteins beforehand, thereby reducing arbitrary factors in such a protein selection step. In contrast, 2D annotation enrichment method is inherently novel since it is the first enrichment method dealing with two 'omics' dimensions simultaneously.

## Competing interests

The authors declare that they have no competing interests.
